# Longitudinal Evaluation Using the Forgotten Joint Score-12 for Double-Bundle Primary Anterior Cruciate Ligament Reconstruction: A Retrospective Observational Study

**DOI:** 10.7759/cureus.70547

**Published:** 2024-09-30

**Authors:** Takuya Sakamoto, Shotaro Watanabe, Manato Horii, Ryu Ito, Seiji Kimura, Satoshi Yamaguchi, Seiji Ohtori, Takahisa Sasho

**Affiliations:** 1 Department of Orthopedic Surgery, Graduate School of Medicine, Chiba University, Chiba, JPN; 2 Center for Preventive Medical Sciences, Chiba University, Chiba, JPN; 3 Department of Orthopedic Surgery, College of Liberal Arts and Sciences, Graduate School of Global and Transdisciplinary Studies, Chiba University, Chiba, JPN

**Keywords:** anterior cruciate ligament (acl) reconstruction, ceiling effect, forgotten joint score, hop test, knee joint stability

## Abstract

Introduction

There are several postoperative evaluation methods for anterior cruciate ligament reconstruction (ACLR), and patient-reported outcome measurement (PROM) is one of the most important for evaluating postoperative clinical results. However, conventional PROMs have a high risk of a ceiling effect at one and two years postoperatively and do not accurately reflect functional improvement over time. Therefore, a longitudinal evaluation using PROM with a low risk of ceiling effect is necessary. The forgotten joint score-12 (FJS) was developed for clinical evaluation after arthroplasty. It is considered an evaluation method after arthroplasty with a low risk of demonstrating a ceiling effect. However, few studies have used the FJS as an evaluation method after ACLR, particularly those tracking changes over time. This study aimed to longitudinally evaluate FJS at one and two years after ACLR.

Methods

This is a retrospective observational study of postoperative patients using existing data and a questionnaire-based survey. This study included patients who underwent primary double-bundle ACLR between August 2017 and August 2021. We compared the FJS, knee injury and osteoarthritis outcome score (KOOS), and Lysholm knee scale (LKS) scores at one and two years post-surgery using the Wilcoxon signed-rank test. The ceiling effect for each PROM was calculated at one and two years post-surgery. A ceiling was defined as obtaining a perfect score in PROMs, and the risk of demonstrating a ceiling effect was the percentage of perfect scores among all cases. The risk of demonstrating a ceiling effect for each PROM was compared using the McNemar test. To identify factors influencing FJS one-year post-ACLR across all cases, multivariate linear regression analysis was conducted for the FJS.

Results

Finally, 87 patients were included in this study. Fifty-six participants were women, and 31 were men, with an average age of 28.5 ± 11.8 years at the time of surgery and a BMI of 23.2 ± 3.7 kg/m^2^. Fifty-eight patients with meniscus injuries requiring treatment were observed. A total of 54 patients were used to compare the results at one and two years, while 87 patients were included in the multivariate analysis for FJS at one year. The median of FJS at one and two years post-surgery were 90.6 and 95.8, respectively. The FJS showed a statistically significant improvement from one to two years (p = 0.033). Question #12 in FJS showed a significant improvement from the first to the second year postoperatively (1.74 ± 1.46 vs 1.15 ± 1.25 at one year vs two years, respectively; p = 0.0016). FJS had a lower risk of demonstrating a ceiling effect than KOOS ADL at one and two years (FJS vs KOOS ADL; at first year: 20.4% and 48.1%, p < 0.001; at second year: 33.3% and 63.0%, p = 0.0013). There was no difference compared to the other PROMs. According to the multivariate linear regression analysis, predictive factors for higher FJS scores at one year post-surgery were younger age and limb symmetry index of single-leg hop test(SLH-LSI) on the affected side that was close to that of the healthy side (SLH-LSI > 0.9).

Conclusions

The FJS continued to improve over two years after ACLR. The FJS post-ACLR was higher in younger individuals and those with SLH-LSI of 0.9 or higher.

## Introduction

Anterior cruciate ligament (ACL) injury is a common sports injury, and patients with instability or high activity are treated with ACL reconstruction (ACLR) surgery. Post-ACLR outcomes are generally favorable, with 78-98% of professional athletes returning to their pre-injury sports level within one year [1]. Several evaluation methods have been reported to assess the clinical course post-surgery [2-4].

Patient-reported outcome measurement (PROM) is one of the important evaluation methods for postoperative clinical results. The KOOS and LKS are the two most commonly used clinical evaluation tools for knee disorders [5]. Several studies have examined the clinical outcomes after ACLR using KOOS and LKS, and they have reported favorable results [3,6]. KOOS can assess functional limitations of the knee joint in sports and daily activities. KOOS scores are associated with functional assessments after ACLR, such as the hop test and the ability to return to sports [4]. However, KOOS and LKS have a high risk of demonstrating a ceiling effect at one and two years post-ACLR procedure assessments and do not accurately reflect functional improvement over time [7-9]. Therefore, a longitudinal evaluation using PROMs with a low risk of ceiling effect is necessary.

The ceiling effect is an inherent limitation of an assessment tool that occurs when a high proportion of participants in a study achieve the highest possible score, making it impossible to differentiate between scores at the upper limit of the scale. Therefore, a high ceiling effect in subjective assessment methods limits their utility and reduces their content validity.

The forgotten joint score-12 (FJS) was developed by Behrend et al. in 2012 for clinical evaluation after arthroplasty [10]. The FJS consists of 12 questions regarding joint awareness during daily activities aimed at achieving a “forgotten knee” after surgery. It is considered an evaluation method after total knee arthroplasty with a low risk of demonstrating a ceiling effect [11]. However, few studies have used the FJS as an evaluation method after ACLR, particularly those tracking changes over time [2,12].

This study aimed to longitudinally evaluate FJS at one and two years after ACLR, compare its ceiling effect with KOOS and LKS, and investigate factors influencing FJS one year after ACLR. We hypothesized that the risk of demonstrating a ceiling effect of the FJS is lower than that of the KOOS and LKS to evaluate the clinical outcomes after ACLR.

This article was previously posted to the Research Square preprint server on August 18, 2023 [13].

## Materials and methods

This study was a retrospective observational examination of postoperative patients using existing data and a questionnaire-based survey. We included patients who underwent primary ACLR using standardized surgical techniques at Chiba University Hospital, Chiba, Japan, between August 2017 and August 2021. We excluded patients with concurrent ligament injuries, a history of ipsilateral knee joint trauma, a history of contralateral ligament injuries, osteoarthritis, and chronic knee joint diseases such as rheumatoid arthritis. Patients with reinjury within two years and deficiencies in data were also excluded.

The institution’s ethics committee approved the study, and all data were collected anonymously to prevent further treatment of specific patients. The use of anonymous data waived the need to obtain informed consent from patients.

Surgical technique

All patients underwent anatomic double-bundle ACLR using hamstring autografts and fixation devices. TightRope-RT (Arthrex, Naples, FL, USA) or Ultrabutton (Smith and Nephew, Andover, MA, USA) was employed on the femoral side, and 1.3 mm TapeLoop (Arthrex) was used on the tibial side. The hamstring tendon was harvested after an arthroscopic examination of the anteromedial (AM) and anterolateral portals. An adjustable suspensory fixator, either TightRope-RT or Ultrabutton, was utilized on the femoral side. On the tibial side, a 1.3 mm artificial ligament called TapeLoop was employed. The graft tendon was folded in half, and the femoral side fixator was passed through the loop side. The cut ends were sutured with Krackow sutures using TapeLoop. A bony tunnel was created in the anatomical position. Two guidewires were inserted from outside the lateral cortex of the femur to the footprints of the AM and posterolateral (PL) bundles of the ACL using an ACL femoral drill guide. Initially, the ACL guide was set to 100° to insert the guidewire into the AM position. Subsequently, the drill guide was adjusted to 95° to insert the guidewire into the PL position. After confirming that the two wire tips were placed in the desired positions, two bone tunnels were drilled over the guidewires from outside the lateral femoral cortex to allow the passage of a retrograde drill to create 21 mm long sockets. The tibial tunnel was established by adjusting the ACL tibial drill guide to 60° to create the AM tunnel and to 55° to form the PL tunnel [14,15]. After passing through the graft, the knee joint was pre-tensioned by flexing the knee 10 times from 0° to 90° while applying manual tensile force [16]. The AM bundle was affixed at 20° of knee flexion, whereas the PL bundle was secured in full extension by applying a traction force of 40 N to the transplanted tendon. The tibial aspect of the graft was fixed using TensionLoc (Arthrex).

Rehabilitation protocol

All patients followed an identical postoperative rehabilitation protocol. Static quadriceps-strengthening exercises were immediately initiated after surgery. Weight-bearing was limited to 30 kg for the first two weeks postoperatively and then unrestricted at three weeks post-surgery. Patients who underwent meniscus repair had a range of motion limited to 90° of knee joint flexion for up to four weeks after the operation, with no range of motion restrictions after four weeks. Patients without meniscus repair had an unrestricted range of motion training. Jogging was initiated at three months postoperatively, and sports training was progressively resumed, starting at seven months postoperatively, aiming to return to sports activities within 10 months.

Data collection

Demographic data, including age, sex, and body mass index (BMI), were extracted from electronic medical records. At one year postoperatively, knee stability was assessed by orthopedic surgeons. This assessment involved measuring the side-to-side difference in anterior tibial translation using KS Measure (Nippon Sigmax Co., Tokyo, Japan), which quantitatively evaluated the anterior drawing test in an outpatient awake state. The single-leg hop (SLH) test was performed in an outpatient setting, recording three trials on both the affected and healthy sides. Subsequently, the average value for each side was computed. To obtain the limb symmetry index of the SLH test (SLH-LSI), the score for the affected side was divided by that of the healthy side. A cutoff of 0.9 for the SLH-LSI was employed to categorize participants into two groups: those with SLH-LSI >0.9 and those with SLH-LSI ≤0.9 [17]. The FJS, KOOS, and LKS were assessed using identical predetermined questionnaires at one and two years postoperatively.

Ceiling effect

The definition of a ceiling was defined as obtaining a perfect score in PROMs, and the risk of demonstrating a ceiling effect in each PROM is defined as the percentage of cases with a perfect score on PROMs out of all cases.

The calculation method is straightforward: we divided the number of cases with a perfect score by the total number of cases and expressed the result as a percentage. This calculation was performed separately for the FJS, KOOS, and LKS. The ceiling effect for each PROM was calculated at one and two years post-surgery.

Statistical analysis

As primary outcomes, we compared the FJS, KOOS, and LKS scores between the first and second years using the Wilcoxon signed-rank test for each score. To evaluate temporal changes in PROMs, we limited the analysis to cases with PROM data at one and two years post-surgery. We compared individual items of the FJS at one and two years postoperatively using the Wilcoxon signed-rank test. The risk of demonstrating a ceiling effect for each PROM was compared using the McNemar test.

As secondary outcomes, to identify factors influencing FJS one year post-ACLR across all cases, multivariate linear regression analysis was conducted for the FJS with age, sex, BMI, side-to-side difference in anterior tibial translation, presence of meniscal injury, and SLH-LSI groups as independent variables. Statistical analysis was performed using EZR R (version 4.1.2), with p < 0.05 considered statistically significant.

## Results

**Figure 1:**
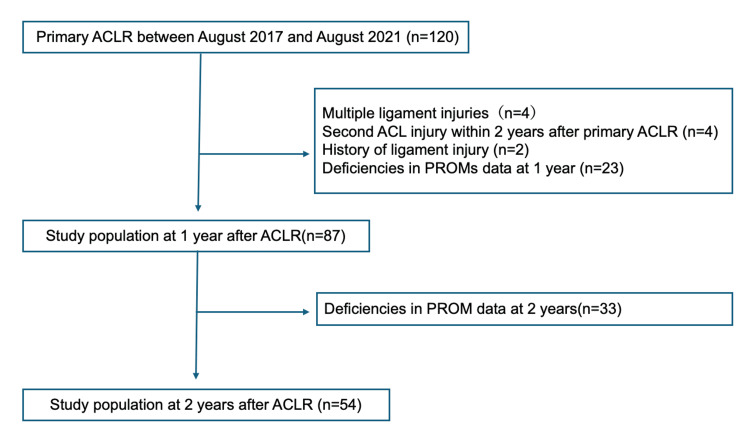
Flowchart of participants throughout the study A total of 120 knees from 120 patients who underwent ACLR were included. Patients with multiple ligament injuries (n = 4), a second ACL injury within two years after ACLR (n = 4), a history of ligament injuries (n = 2), and those with deficiencies in PROMs one year after ACLR (n = 23) were excluded. Thus, 87 patients were included in this study. Patients with missing data for the second year were excluded from the analysis conducted at the two-year postoperatively. ACL, anterior cruciate ligament; ACLR, anterior cruciate ligament reconstruction; PROMs, patient-reported outcome measures

In total, 120 patients underwent primary ACLR. Patients with multiple ligament injuries (n = 4), ACL reinjury within two years after ACLR (n = 4), a history of ligament injuries (n = 2), or missing PROM data at one year postoperatively (n = 23) were excluded. Thus, 87 patients were included in this study (Figure 1).

Participant demographics

Table 1 shows the demographic characteristics of the participants at one and two years post-surgery. At one year, there were 56 female and 31 male participants, with an average age of 28.5 ± 11.8 years at the time of surgery and a BMI of 23.2 ± 3.7 kg/m^2^. Fifty-eight patients had meniscal injuries requiring treatment. At two years post-surgery, there were 54 participants. There were no differences in the demographic characteristics between one and two years.

**Table 1:** Baseline characteristics at the first and second year postoperatively *Data expressed as count (percentage %). ‡Data expressed as mean ± standard deviation. BMI, body mass index; SLH-LSI, limb symmetry index of single-leg hop test

Variables	1 year postoperatively (n = 87)	2 years postoperatively (n = 54)
Sex*		
Male	31 (35.6)	14 (25.9)
Female	56 (64.4)	40 (74.1)
Age (years)‡	28.5 ± 11.3	28.1 ± 11.0
BMI (kg/m^2^)‡	23.2 ± 3.7	23.4 ± 4.0
Meniscus status*		
Intact	29 (33.3)	13 (24.1)
Injury	58 (66.7)	41 (75.9)
SLH-LSI*		
>0.9	56 (64.4)	NA
≦0.9	31 (35.6)	NA

Longitudinal changes in PROMs from one to two years

Every PROM showed significant improvement from one to two years. The median of FJS at one and two years post-surgery were 90.6 and 95.8 (p = 0.033), respectively. Every subscale of the KOOS also showed significant improvement from one to two years (symptoms: 92.9 vs 96.4, p < 0.001; pain: 94.0 vs 97.2, p = 0.009; ADL: 98.5 vs 100.0, p = 0.024; sports/rec: 85.0 vs 95.0, p < 0.001; QOL: 78.2 vs 87.5, p < 0.001). LKS between one and two years were 94.0 vs 95.0, p = 0.003) (Table 2).

**Table 2:** Longitudinal patient-reported outcome measures at one and two years after ACLR Data expressed as median and IQR. FJS, forgotten joint score-12; KOOS, knee injury and osteoarthritis outcome score; LKS, Lysholm knee scale

Postoperative	1 year	2 years	p-value
Total FJS	90.6 (78.2-97.9)	95.8 (87.5-100)	0.033
KOOS			
Symptoms	92.9 (78.6-96.4)	96.4 (60.7-100)	<0.001
Pain	94.0 (88.9-100)	97.2 (91.7-100)	0.009
ADL	98.5 (96.0-100)	100.0 (98.5-100)	0.024
Sports/rec	85.0 (80.0-95.0)	95.0 (85.0-100)	<0.001
QOL	78.2 (68.8-98.5)	87.5 (75.0-100)	<0.003
LKS	94.0 (86.3-100)	95.0 (91.0-100)	0.003

Changes in the subscales of the FJS-12

Only question #12 in FJS showed a significant improvement from the first to the second year postoperatively (1.74 ± 1.46 vs 1.15 ± 1.25 at one year vs two years, respectively; p 0.0016). Other items tended to improve, but no significant differences were observed (Table 3).

**Table 3:** Total and subscales of FJS-12 at one and two years after ACLR Data were represented as mean ± standard deviation. The FJS consists of 12 questions with five choices (from 0 to 4; 0 means the best score) and is scored using a format with the raw scores transformed onto a 0-100 point scale (100 means the best score). ACLR, anterior cruciate ligament reconstruction; FJS, forgotten joint score-12

Postoperative	1 year	2 years	p-value	
FJS 1	0.24 ± 0.73	0.09 ± 0.35	0.16	
FJS 2	0.44 ± 0.86	0.24 ± 0.64	0.14	
FJS 3	0.48 ± 0.88	0.26 ± 0.59	0.13	
FJS 4	0.22 ± 0.74	0.07 ± 0.33	0.17	
FJS 5	0.28 ± 0.76	0.15 ± 0.53	0.30	
FJS 6	0.56 ± 0.95	0.44 ± 0.95	0.50	
FJS 7	0.63 ± 1.01	0.46 ± 0.82	0.33	
FJS 8	0.72 ± 1.04	0.52 ± 0.88	0.17	
FJS 9	0.74 ± 1.05	0.56 ± 0.96	0.059	
FJS 10	0.37 ± 0.76	0.28 ± 0.79	0.50	
FJS 11	0.63 ± 1.09	0.44 ± 0.84	0.18	
FJS 12	1.74 ± 1.46	1.15 ± 1.25	0.0016	

Ceiling effects

**Figure 2:**
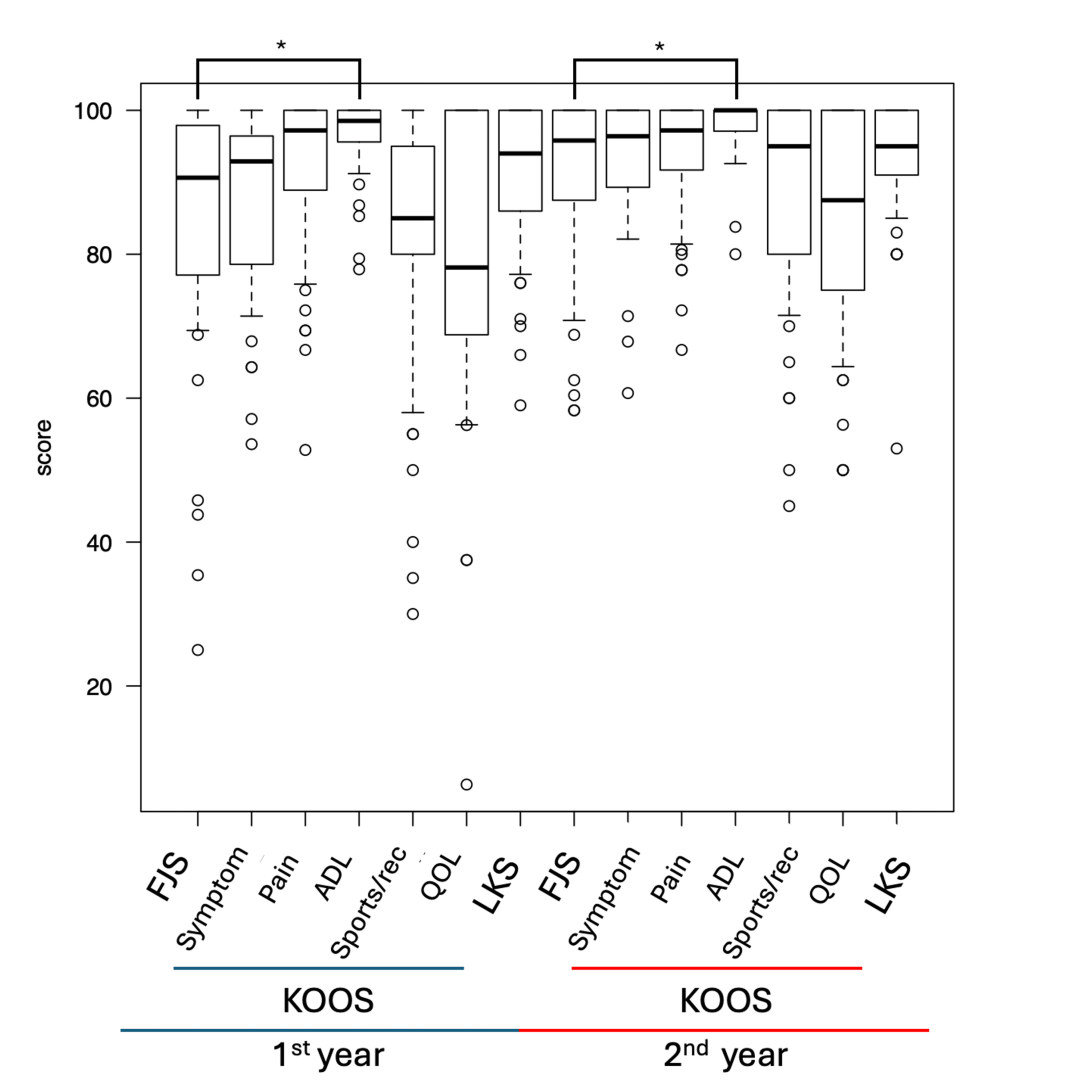
The box-plot diagram for each score *p < 0.01 FJS, forgotten joint score-12; KOOS, knee injury and osteoarthritis outcomes score; ADL, activities of daily living; QOL, quality of life; Rec, recreation; LKS, Lysholm knee scoring scale

The ceiling effect for the FJS was 20.4% at one year and 33.3% at two years postoperatively. For the KOOS, the ceiling effect was as follows: 22.2% for symptoms, 27.8% for pain, 48.1% for ADL, 20.4% for sports, and 25.9% for quality of life (QOL) at one year postoperatively, and 44.4% for symptoms, 44.4% for pain, 63.0% for ADL, 38.9% for sports, and 35.2% for QOL at two years postoperatively. The ceiling effect for LKS was 27.8% at one year and 44.4% at two years postoperatively. Figure 2 shows a box-plot diagram for each score. FJS had a lower risk of demonstrating a ceiling effect than KOOS ADL at one and two years (FJS vs KOOS ADL; at first year: 20.4% and 48.1%, p < 0.001; at second year: 33.3% and 63.0%, p = 0.0013). There was no difference compared to the other PROMs.

Factors influencing the FJS-12 one year after ACLR

Table 4 presents the results of the multivariate linear regression analysis. The variance inflation factors were all less than 4, indicating no multicollinearity among the variables. According to the multivariate linear regression analysis, the FJS score at one year post-surgery decreased with increasing age (estimated regression coefficient = −0.49, t = −2.32, p = 0.023). Additionally, a predictive factor for higher FJS scores at one year post-surgery was an improvement in the affected side SLH-LSI to a value close to that of the healthy side (SLH-LSI >0.9) (estimated regression coefficient = 15.37, t = 3.16, p = 0.002).

**Table 4:** A multiple linear regression analysis to predict FJS one year after ACLR ACLR, anterior cruciate ligament reconstruction; FJS, forgotten joint score-12; BMI, body mass index; SLH-LSI, limb symmetry index of single-leg hop; CI, confidence interval

Variables	Estimated coefficient	Standard deviation	t-value	95% CI	p-value
Intercept	75.97	16.48	4.61	43.07, 108.87	<0.01
Age (years)	-0.47	0.20	-2.35	-0.87, 0.07	0.02
Sex (male＝0, female = 1)	-3.26	4.87	-0.67	-13.01, 6.49	0.51
BMI (kg/m^2^)	0.52	0.66	0.79	-0.80, 1.84	0.43
Side-to-side difference of tibial translation (mm)	-1.39	0.91	-1.54	-3.21, 0.42	0.13
SLH-LSI (<0.9 = 0, >0.9 = 1)	15.37	4.87	3.16	5.66, 25.09	>0.01
Meniscus status (intact = 0, injury = 1)	1.03	4.96	0.21	-8.86, 10.93	0.84

## Discussion

The most important aspect of this study is the longitudinal evaluation of clinical outcomes after ACLR using the FJS. Previous studies have reported a low risk of demonstrating a ceiling effect in longitudinal assessments of FJS after joint arthroplasty. Cross-sectional studies evaluating FJS after ACLR have also shown that FJS has a lower ceiling effect compared to other PROMs, supporting the hypothesis that the risk of ceiling effect would be low. In this study, the risk of a ceiling effect in FJS was lower than in KOOS ADL. KOOS ADL assesses daily living activities, which may explain the high ceiling effect observed in postoperative evaluations after ACLR. Although FJS also focuses on daily activities, its inclusion of a sports-related question (question 12) and elements of psychological evaluation might have contributed to the observed differences. However, FJS was not significantly different from other PROMs, and the FJS scores in our study were relatively high compared to previous reports.

Unlike previous cross-sectional studies, the strength of this study lies in its longitudinal evaluation of FJS, allowing us to report temporal changes. The results showed an improvement from one to two years postoperatively, similar to other PROMs, with a statistically significant improvement observed only in question #12 in FJS. This may be because the FJS was initially developed for joint arthroplasty patients, who are generally older and less active than ACLR patients. Consequently, ACLR patients might not perceive discomfort during daily activities, as captured by questions other than question #12 in FJS. However, clear discomfort during sports activities was noted. This finding highlights the need for a specific tool that evaluates the psychological aspects of ACLR during sports activities, suggesting the potential need for an ACLR-specific forgotten joint assessment tool in the future.

This study also investigated the factors influencing the overall FJS score one year postoperatively. The results identified age and SLH-LSI as significant factors.

Age has been previously reported as a factor that may influence postoperative outcomes after ACLR. Observational studies have indicated that older age is associated with lower KOOS, LKS, and IKDC scores after ACLR. Recent studies comparing younger and middle-aged groups have reported that postoperative outcomes in the middle-aged group are comparable to those in the younger group [18-20]. On the other hand, a retrospective study reported that ACLR improves pain during activity; however, it does not improve pain at rest in middle-aged adults [21]. In our study, we found that FJS scores decreased with increasing age.

SLH-LSI has previously been reported as a factor influencing the return to sports after ACLR [22-24]. In a study involving patients who had not returned to their pre-injury sports level one year post-ACLR, SLH-LSI was better in the group that returned to sports two years postoperatively [1]. In our study, question #12 in FJS, which assesses discomfort in the knee during sports, showed a significant improvement from the first to the second year. Similar to various sports activities, SLH involves not just isolated muscle strength but also complex movements such as jumping and stopping, suggesting the importance of psychological evaluation during sports after ACLR. Recently, ACL-RSI, a PROM focusing on the psychological aspects of ACLR, has been reported as a return-to-sports criterion, underscoring the growing recognition of psychological evaluation after ACLR.

Our study had several limitations. First, possible selection bias could arise due to the large number of patients who interrupted outpatient visits during the two-year follow-up after surgery, as well as the small sample size. The follow-up rate of outpatients decreased during this period due to the coronavirus disease 2019 pandemic. However, a comparison between the 33 patients who interrupted outpatient visits within two years and the 54 patients who remained showed no significant differences in patient demographics or PROMs at one year post-surgery. Therefore, the effect of selection bias was expected to be minimal. Our study is one of the few to investigate the association between ACLR and FJS. Second, it is unclear whether the one- and two-year postoperative time points are truly appropriate for evaluating the ceiling effect. One of the limitations of this study is that the ceiling effect beyond two years postoperatively was not investigated. However, it is generally considered that clinical outcomes reach a plateau by two years postoperatively, making accurate evaluation during this period particularly important. Therefore, the lack of data beyond two years is unlikely to be a significant drawback. Third, patient-reported outcomes are subject to the effects of translation. Although the validity of the Japanese version of FJS has been established for joint arthroplasty, our study involved younger patients, raising the possibility that the ceiling effect of the Japanese version of FJS may vary with age. Finally, The inclusion of only double-bundle reconstruction also limits the generalizability of our findings.

## Conclusions

This study was the first to investigate the longitudinal clinical outcomes of ACLR using FJS. The FJS demonstrated continued improvement over the two years after ACLR (90.6 and 95.8, p = 0.033), and question #12 in FJS showed a significant improvement from the first to the second year postoperatively (1.74 ± 1.46 vs 1.15 ± 1.25 at one year vs two years, respectively; p = 0.0016).

The risk of demonstrating a ceiling effect of FJS was lower than KOOS ADL at both one and two years (FJS vs KOOS ADL; at first year: 20.4% and 48.1%, p < 0.001; at second year: 33.3% and 63.0%, p = 0.0013), but there was no difference compared to the other PROMs.

In younger individuals and those with an SLH-LSI of 0.9 or higher, FJS post-ACLR scores were higher.
